# Is liquid biopsy a cost-effective method to diagnose Burkitt Lymphoma in children and young adults? A health economic evaluation in Tanzania

**DOI:** 10.1186/s12916-026-04694-2

**Published:** 2026-02-21

**Authors:** Jingjing Jiang, Liz Morrell, Malale Tungu, William F. Mawalla, Clara Chamba, Lulu Chirande, Heronima J. Kashaigili, Elifuraha Mkwizu, Paul S. Ntemi, Godlove Sandi, Kristin Schroeder, Anna Schuh, George M. Ruhago, Sarah Wordsworth, AI-REAL Consortium, Jingjing Jiang, Liz Morrell, George M. Ruhago, Caroline Achola, Pamela Atim, Adam Burns, Clara Chamba, Lulu Chirande, Faraja Chiwanga, Anthony Cutts, Helene Dreau, Claire El Mouden, Edrick M. Elias, Philomena Goodluck, Oliver Henke, Kieran Howard, Daisy Jennings, Emmanuel Josephat, Atukuzwe Kahakwa, Hadija Kaliisa, Jacqueline Kamanga, Ismail D. Legason, Mihaela Leonte, Laura Lopez Pascua, Erick Magorosa, Salama Mahawi, Priscus Mapendo, William F. Mawalla, Sam M. Mbulaiteye, Daniel Mbwambo, Elifuraha Mkwizu, Leah Mnango, Alex Mremi, Hadija Mwamtemi, Liberata Mwita, Martin D. Ogwang, Isaac Otim, Kate Ridout, Godlove Sandi, Raphael Sangeda, Patricia Scanlan, Kristin Schroeder, Anna Schuh, Rehema Shungu, Paul Shadrack Ntemi, Malale Tungu, Dimitris Vavoulis, Sarah Wordsworth

**Affiliations:** 1 Health Economics Research Centre, Nuffield Department of Population Health, University of Oxford, Oxford, UK; 2 Wolfson Institute of Population Health, Queen Mary University of London, Charterhouse Square, London, UK; 3 Department of Development Studies, School of Public Health and Social Sciences, Muhimbili University of Health and Allied Sciences, Dar es Salaam, Tanzania; 4 Department of Haematology and Blood Transfusion, Muhimbili University of Health and Allied Sciences, Dar es Salaam, Tanzania; 5 Department of Oncology, Bugando Medical Centre, Mwanza, Tanzania; 6 Department of Internal Medicine, School of Medicine, KCMC University, Moshi, Tanzania; 7 Department of Paediatrics and Child Health, Muhimbili National Hospital, Dar es Salaam, Tanzania; 8 Department of Pediatrics and Duke Global Health Institute, Duke University, Durham, NC USA; 9 Department of Oncology, University of Oxford, Oxford, UK; 10 National Institute for Health and Care Research Oxford Biomedical Research Centre, University of Oxford, Oxford, UK

**Keywords:** Sub-Saharan Africa, Tanzania, Burkitt Lymphoma, Paediatric oncology, Diagnosis, Liquid biopsy, Histopathology, Sequencing panel, Cost-effectiveness, DALY

## Abstract

**Background:**

Burkitt Lymphoma (BL) is a prevalent and highly aggressive childhood cancer in sub-Saharan Africa (SSA). Outcomes are poor, due in part to delays or inaccuracies in diagnosis. Liquid biopsy (sequencing circulating tumour DNA from blood) is an alternative approach that is non-invasive and offers potential for a highly specific, rapid diagnosis, which could improve outcomes through earlier access to correct treatment. Diagnosis by liquid biopsy has undergone validation in SSA. However, evidence on its economic value is needed, especially in limited-resource settings. We present a cost-utility analysis comparing liquid biopsy with conventional local histopathology for the diagnosis of paediatric BL in Tanzania.

**Methods:**

A cost-utility model was constructed to compare the costs and effects of the alternative diagnostic approaches from a Tanzanian healthcare provider perspective, over a lifetime horizon. For the liquid biopsy, we assumed testing at the patient’s first contact with healthcare, resulting in earlier diagnosis. Resource use and outcome data were taken from the Aggressive Infection-Related East Africa Lymphoma study. Health outcomes were measured in Disability-Adjusted Life Years (DALYs). Costs and outcomes were discounted at 5%. Probabilistic and univariate sensitivity analyses and scenario analysis were conducted.

**Results:**

Diagnosis by liquid biopsy results in a reduced burden of 9.4 DALYs per patient compared to 10.5 for histopathology (difference 1.11 DALYs, 95% confidence interval 0.13–2.06), and per-patient costs are $1978 higher (95% CI $1299–2840), due to greater diagnosis costs. The incremental cost-effectiveness ratio (ICER) is $1778 per DALY averted. Probabilistic sensitivity analysis indicates that liquid biopsy is more likely to be cost-effective than pathology, at any threshold above $1890/DALY averted. The ICER is sensitive to assumptions regarding the extent of disease advancement at the earlier diagnosis, and to discount rate and diagnosis costs.

**Conclusions:**

Diagnosis by liquid biopsy could be cost-effective when used to accelerate diagnosis of BL in children and young adults in Tanzania, depending on when it is implemented in the patient journey, model assumptions and the decision threshold chosen.

**Trial registration:**

Pan African Clinical Trials Registry: PACTR202204822312651, registered on 14th April 2022.

**Supplementary Information:**

The online version contains supplementary material available at 10.1186/s12916-026-04694-2.

## Background

Approximately 400,000 children are diagnosed with cancer annually worldwide, with over 85% of cases occurring in low- and middle-income countries (LMICs) [1]. The survival rate for children with cancer exceeds 80% in high-income countries (HICs), but is less than 30% in LMICs [2]. Childhood cancers are a significant healthcare burden especially in sub-Saharan Africa (SSA), where Burkitt Lymphoma (BL) is one of the most prevalent childhood malignancies [3].

BL is an aggressive non-Hodgkin lymphoma, notable by its rapid growth, which predominantly affects the jaw, central nervous system and abdominal organs, and is fatal without treatment [4]. It was historically classified into three types—endemic, sporadic and immunodeficiency-associated [4, 5]. Endemic BL is the most prevalent type in SSA, accounting for 50% to 70% of all paediatric malignancies [6]. Within the region, annual incidence ranges from 0.5 to 19.3 cases per million [7, 8], is particularly high in the malarial belt of Africa and is strongly associated with early Epstein-Barr virus (EBV) infection [9]. Endemic BL mainly affects children, with peak incidence at age 4–7, and twofold higher rates in males [10, 11]. Presentation is commonly with a facial or abdominal mass [10] and the majority present at an advanced stage of disease (stages III or IV of common staging systems) [11, 12]. In contrast, the sporadic form is found worldwide, including HICs [10]. It has a lower incidence of 2–3 cases per million [8], and occurs across a broader age range including adults, again predominantly in males [10, 13]. Association with EBV is only 15%–30% of patients, and presentation is typically with an abdominal mass and symptoms [10].

The cure rate of BL is over 90% in HICs, attributed to routine molecular diagnostic testing for definitive mutations, and intensive chemotherapy combined with supportive care to manage complications [14, 15]. However, in SSA, the survival rate is lower than 50% [16]. Reasons for this poor outcome are multifaceted, largely related to health system constraints. In Tanzania, only three centres treat children with cancer [11], and these are accessed via a hierarchical referral system from lower-level facilities (health centres, regional hospitals). Inefficient referral [17] and the disincentive of travel (cost and time) to a non-local cancer centre [17, 18] result in delayed presentation at a cancer centre; this delay from first presentation to assessment by an oncologist has been estimated variously as a median of 89 days (paediatric cancers), 37 days (children and young people with lymphoma) and 23 days (children and young people with BL) [18, 19], with longer delays and higher out-of-pocket payments for socially disadvantaged and rural patients [17]. The resultant advanced disease at diagnosis [12] leads to poorer outcomes [20]. There are risks of further delay due to availability of clinicians (there are only 6 paediatric oncologists in Tanzania [12]) and issues of drug supply [21]. Treatments are typically less intensive than in high-income countries, due to limited supportive care, with resultant lower survival [14, 16]. Although cancer treatment is funded in Tanzania, families may find it difficult to continue treatment due to costs of accommodation and food; to help alleviate this deterrent, funding is available through donor organisations, and several centres provide free hostel accommodation [22]. Further, limitations in imaging and availability of bone marrow samples mean not all patients receive complete staging to allow risk-adjusted treatment [11, 12, 23].

Regarding diagnosis, the current standard approach for BL in SSA involves invasive biopsy procedures with histopathological analysis. However, workforce limitations are also evident here, with limited numbers of surgeons and pathologists (estimated as 1 per million people [24]) to perform and interpret biopsies [6]. Challenges in carrying out high-quality pathology result from reagent supply issues [25], variable implementation of quality standards [26] and limited availability of automated systems needed for reproducible histochemistry [6, 14, 19]. This leads to misdiagnoses and delays [14], and patients starting treatment before a full immunohistochemical diagnosis is available [11, 19]. Reported pathology turnaround times vary; examples include a median of 28 days for lymphoma patients in Tanzania and Uganda [19], and a median of 71 days among cancer patients in Malawi [27].

Liquid biopsy is a non-invasive diagnostic method using DNA sequencing to detect tumour-associated mutations in circulating tumour DNA (ctDNA) isolated from body fluids, particularly peripheral blood [6, 28]. The approach overcomes the limitations of traditional tissue sampling such as tumour access and tumour heterogeneity [29] and can potentially be used for real-time disease monitoring [28]. Because BL has a specific and well-characterised genetic basis [5], it is an ideal candidate for diagnosis by liquid biopsy. In this context, liquid biopsy offers precise diagnosis, requiring a simple blood draw rather than invasive biopsy, and with a potentially shorter turnaround time than current histopathology [6, 30].

The Aggressive Infection-Related East Africa Lymphoma (AI-REAL) project (2019–2024) established the first human diagnostic DNA sequencing capability in east Africa, developed a BL detection method using targeted sequencing of relevant regions of the tumour genome (known as a targeted panel) and clinically validated its use for diagnosing BL in children and young adults [30]. In addition to obtaining evidence on the clinical benefits of alternative diagnostic approaches, it is important to provide evidence on the potential economic benefits of this diagnostic for BL, especially in limited-resource settings. As such, as part of the AI-REAL project we conducted a cost-effectiveness analysis to evaluate liquid biopsy compared to the standard diagnostic approach for BL in children and young adults in Tanzania.

## Methods

The AI-REAL clinical validation of liquid biopsy in BL diagnosis was conducted between May 2019 and June 2023 in four hospital sites in Tanzania (Muhimbili National Hospital, Dar-es-Salaam; Kilimanjaro Christian Medical Centre, Moshi; Bugando Medical Centre, Mwanza) and Uganda (St. Mary’s Hospital Lacor, Gulu) [30]. The study recruited patients aged 3 to 30 years based on clinical suspicion of lymphoma. Following diagnosis by histopathology, patients received chemotherapy according to local protocols. Liquid biopsy (blood) samples taken concurrently were analysed subsequently and compared to histopathology to determine concordance of the diagnoses.

### Model design

The intervention is liquid biopsy (blood sample, ctDNA extraction and sequencing), compared with the standard diagnostic approach (tissue biopsy with histopathological analysis). The evaluation was conducted from a Tanzanian healthcare provider perspective, incorporating direct medical costs for diagnostics and treatments. We chose to concentrate on a single decision-making context and selected Tanzania where our largest study site and partner university are located, and where local diagnostic costing was conducted; however, we retained data on Ugandan patients in data from AI-REAL to maximise use of available evidence. A lifetime time horizon was applied to capture all relevant costs and outcomes, which were discounted at 5% per annum as recommended for global health evaluations [31]. Reporting adheres to the CHEERS guidance for health economic evaluation [32] (Additional File 1).

**Fig. 1:**
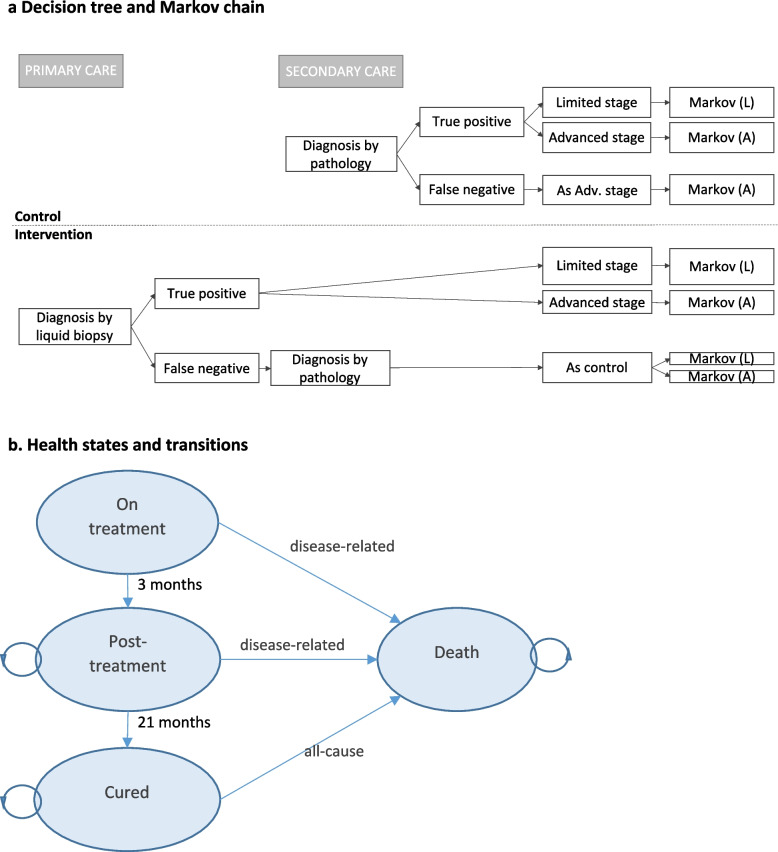
Model structure. **a** Decision tree and Markov chains.** b** Health states and transitions. *Primary care*, first contact with healthcare, such as a healthcare centre, local or regional hospital; *Secondary care*, referral-level cancer centre; *Markov*: cohort enters Markov model parameterised for limited or advanced stage patients; *L*, limited stage; *A,* advanced stage. Patients remain in the on-treatment state for one model cycle (3 months) reflecting the typical duration of first treatment, then transition to the post-treatment state. All treatment costs other than end-of-life care are modelled as being incurred during this first cycle

A decision-analytic model combining a decision tree with a Markov model (Fig. 1) was constructed in Microsoft Excel. The model followed a cohort of patients aged 10 years (mean age from AI-REAL) diagnosed with BL. The control arm reflected current diagnostic practice for BL patients. Following diagnosis and disease staging, patients entered a Markov chain parameterised for either limited or advanced stage disease. Patients who were initially misdiagnosed (BL false negatives, FN) were assumed to experience a delay in starting appropriate treatment, during which time their disease would progress; as a simplifying assumption, we assumed these patients would have reached an advanced stage of disease on starting correct treatment.

By design, the validation study did not use the experimental liquid biopsy diagnostic to make treatment decisions; hence, we did not have direct data on patients diagnosed using liquid biopsy. Instead, we constructed a hypothetical liquid biopsy arm taking advantage of the ease of collecting the blood sample, which can be taken by a competent professional in a local health centre. We modelled a scenario where this sample was taken at the patient’s first healthcare contact, such as a primary healthcare centre or a regional hospital. We then assumed that with the diagnostic process starting earlier, fewer patients would have reached an advanced stage of disease by the time they complete diagnosis and staging, compared to the current 60% advanced stage found in AI-REAL. AI-REAL analysis indicates the gap between first contact and presenting at a cancer centre is 3–4 weeks for BL patients [19]; clinical opinion suggested that in this interval, up to half of the limited stage patients would progress to advanced stage. We therefore chose to model this earlier-tested population as consisting of 60% limited-stage patients as a mid-range estimate (i.e. assuming that under current practice, a third of these have progressed to advanced stage before presenting at a cancer centre).

FNs by liquid biopsy were modelled as subsequently following the current histopathology pathway, on the same timing and with the same stage distribution as the control arm. We assumed 81% sensitivity for the liquid biopsy based on AIREAL validation data, at a specificity of 94%; this is consistent with initial published analyses with sensitivities in the range 80%–92% [33].

The control arm assumed that all biopsies are amenable to diagnosis by histopathology, regardless of cancer type. The targeted panel, however, is specifically designed to diagnose BL, so only a proportion of those tested will receive a definitive diagnosis. To account for this, we applied a multiplier on the cost of liquid biopsy, reflecting the number needed to test (NNT) to diagnose one BL patient. In AI-REAL, among patients with suspected lymphoma, 25% were diagnosed as BL. We therefore assumed a NNT multiplier of four as the base case in our model.

The Markov model had 4 health states, with a model cycle of 3 months reflecting the duration of initial treatment. Patients entered the treatment state for one cycle, then transitioned to post-treatment. The natural history of the disease is such that relapse and death from disease typically occur during the first 2 years; in line with other studies, we therefore assumed that after 2 years patients enter a post-disease state where they experience no additional morbidity or mortality compared to their age cohort [34]. Patients transitioned to the dead state from any of the other states. As the disease is aggressive, based on clinical opinion, we assumed that death from disease happens within a model cycle and imposed both care costs and a quality-of-life reduction in that cycle. A half-cycle correction was implemented.

### Model inputs

Model inputs are shown in Table 1 and were derived from data for 86 study patients who were diagnosed with BL. Patients’ disease stage was classified as limited (stages I/II, *n* = 34) or advanced (stages III/IV, *n* = 52), using the St Jude’s staging system, and model parameters were derived for each group.

**Table 1:** Model inputs

**Parameter**		**Value**	**Distribution**	**Sensitivity analysis**	**Source**
Discount rate (annual)	Costs and outcomes	5%	··	3.5%, 10%	Haacker et al. 2020 [31]
Overheads		20%	··	15%, 25%	Denburg et al. 2019 [34], Renner et al. 2018 [35]
Number needed to test	(Liquid biopsy)	4	··	2–6	AI-REAL
Proportion of false negatives	Pathology	0.221	··	0.13, 0.31 (95% CI)	AI-REAL
	Liquid biopsy	0.187	··	0.08, 0.22	Chamba et al. 2025 [33], AI-REAL (unpublished)
Proportion of patients at limited stage	Pathology	0.396	··	0.3, 0.5 (95% CI)	AI-REAL
	Liquid biopsy	0.6	··	0.4–0.8	Clinical opinion
Age of cohort		10 years	··		AI-REAL
**Outcomes:**		**Value (SE)**			
BL DALY weights	Active disease	0.288 (0.0526)	Beta	··	Salomon et al. 2015 [36]
	Terminal disease	0.540 (0.0105)	Beta	··	
	Post treatment	0.049 (0.0791)	Beta	··	
Survival parameters	Constant	− 2.702 (0.4186)	Normal	··	AI-REAL
	Coefficient: advanced	0.951 (0.4101)	Normal	··	
	Gamma	0.500 (0.0781)	Normal	··	
Adverse event probabilities *			Beta	··	AI-REAL
**Costs:**		**TZS (SE)**			
Diagnosis	Pathology	413,933	Gamma	± 20%	Morrell et al. [37]
	Liquid biopsy	1,308,450			
Chemotherapy: drugs	Limited	2,777,813 (361,404)	Gamma	± 20%	AI-REAL
	Advanced	2,146,124 (300,775)			
Chemotherapy: non-drug	Limited	1,598,578 (96,678)	Gamma	± 20%	AI-REAL
	Advanced	1,474,471 (82,376)			
Adverse event treatment*			Gamma	··	AI-REAL
Follow-up (per quarter)	Year 1	87,581 (17,516)	Gamma	··	AI-REAL
	Year 2	23,790 (4758)			
	Years 3–5	11,895 (2379)			
End-of-life treatment		166,380 (41,766)	Gamma	··	AI-REAL

Model inputs, showing their distributions used for probabilistic sensitivity analysis, the range of values used in univariate sensitivity analysis, and the source

^*^See Additional File 4: Table 3 for probabilities of each adverse event type by disease stage and their respective per-incident cost

*TZS *Tanzanian Shillings, *95% CI* 95% confidence interval, *SE *standard error

### Outcomes

Health outcomes were measured in Disability-Adjusted Life Years (DALYs). DALYs provide a comprehensive measure of disease burden that captures both morbidity and mortality impact of the disease and its treatment. Although developed for measuring disease burden rather than for economic evaluation, the DALY is commonly used in global health evaluations [34, 38, 39], including in the WHO-CHOICE generalised cost effectiveness evaluation [40], and the literature on cost-effectiveness thresholds in global health is couched in DALY terms [41].

Overall survival data by stage were taken from AI-REAL (all causes of death), and fit to a range of commonly used distributions. The Weibull model was chosen based on fit statistics and visual inspection of the fit to the Kaplan–Meier plots over 24 months (Additional File 2: Table 1). The resultant transition matrix is shown in Additional File 2: Table 2. After 2 years, we assume age-specific all-cause mortality for the population, taken from the current Global Burden of Disease (GBD) Study data for Tanzania [42] (Additional File 3). The years of life lost (YLL) component of the DALY was discounted [43], such that for example, a child dying at the age of 10 with a remaining life expectancy of 65 years accrues a burden of ~ 20 YLL.

Disability weights for BL to calculate years living with disability (YLD) were taken from the GBD study [36]. Age-specific disability weights for eastern SSA were taken from GBD 2019 [44] (Additional File 3).

#### Costs

##### Resource use

Chemotherapy drug dosage for each regimen was taken from local treatment protocols [45] (Additional File 4: Table 1). Per-patient drug consumption was then derived using AI-REAL data on treatment regimen and number of treatment cycles. No drug wastage was assumed. Other resources used in treatment (‘non-drug’ costs in Table 1) included laboratory investigations, imaging, supportive treatments such as fluids, hospital visits and bed-nights, and consultations; treatment-cycle-specific resource use was derived from local treatment protocols with clarifying input from AI-REAL clinicians (Additional File 4: Table 2). These were then applied per-patient based on their cycle count. Incidence of grade 3–4 adverse events was taken from AI-REAL; resource use for treatment was derived from local protocols and clinical expertise (Additional File 4: Table 3). Follow-up costs assumed per-protocol monthly consultations in the first year (every third in person, others by telephone), half-yearly in year 2 and annually for years 3–5. AI-REAL data and clinical expertise were used to derive a typical end-of-life treatment and number of bed-nights for patients dying of BL (Additional File 4: Table 4); no cost of dying was included for non-disease deaths.

##### Unit costs

Costs for diagnosis by pathology and liquid biopsy were taken from a published microcosting [37], using the liquid biopsy cost for 900 samples per year based on an estimated 171 incident cases per year in Tanzania [8] and assuming four samples tested per case identified. Costs of medications, investigations and hospital visits were obtained from AI-REAL hospital sites in Tanzania, as were clinical salaries. In the absence of specific institutional data, overheads were included as 20% of all medical costs for both arms [34, 35]. Cost information was collected in local currency, during 2023, with any values from other years converted to 2023 values. Results are also shown in dollars ($ = 2595 TZS, October 2024).

### Cost-effectiveness

Cost-effectiveness is reported as the incremental cost effectiveness ratio (ICER), or incremental cost per DALY averted. To reflect the diverse approaches that have been applied to defining an ICER considered cost effective, we compared the ICER to a range of commonly used thresholds: 3 × per-capita GDP and 1 × GDP as discussed by the WHO-CHOICE programme (although with the caveat that these should not be used as a standalone threshold for decision-making [46]), and the opportunity cost approach of Ochalek et al. [41], converted to local currency and inflated to 2023. These give 2023 threshold values of $3633, $1211 and $411, respectively, interpreted in this paper as the amount the health system is willing to pay to avert one DALY.

#### Sensitivity analysis

One-way sensitivity analysis was conducted to explore the effect of the main parameters on cost effectiveness (Table 1). The values were varied by ± 20%, or used a range from observed data where available. In addition, scenario analyses were conducted to explore the effect of key assumptions in the model. Firstly, to test the assumption that 1 in 4 samples sent for liquid biopsy will be BL patients, the NNT multiplier was varied from 2 to 6; for multipliers of 2 and 3, we used the (slightly higher) per-patient liquid biopsy cost estimate for 300 samples per year [37]. A second key assumption was the proportion of patients who would have limited-stage disease at the earlier diagnosis; we tested 40%–80% (equivalent to no progression, and 50% of limited-stage patients progressing, respectively). Third, we explored the assumption that death from disease occurs in the first 2 years, by extending the risk of death from BL to 3, 4 and 5 years; additional deaths in the extended period incurred the same terminal disease weight and disease-related end-of-life costs as the base case, in the cycle in which they died. Fourth, we relaxed the assumption that FN patients by histopathology have the prognosis of advanced-stage patients, by retaining their original staging in the model. Fifth, we evaluated the effect of availability of liquid biopsy diagnosis, from 20 to 100%, by combining costs and outcomes in proportion, having adjusted liquid biopsy costs for throughput [37]. In addition, given the aggressive nature of the disease, we explored the effect of a shorter model cycle, reducing the cycle length to 1 week over the 2-year period of disease risk (all diagnosis and treatment costs incurred at the start as in the base case). Finally, AI-REAL included patients from both Tanzania and Uganda; as the economic analysis takes a Tanzanian perspective, we tested a scenario using data from only Tanzanian patients (*n* = 46).

In addition to routine chemotherapy, eligible AI-REAL patients were offered targeted treatment with rituximab, which is effective and widely used to treat lymphoma in high-resource countries [15]. As it is not in routine use in SSA, we created an approximation to a ‘rituximab-free’ scenario; all costs for rituximab were removed, and outcomes were adjusted by re-estimating a Weibull survival model for all patients by both stage and rituximab status (Additional File 5: Fig. 1, Table 1).

Probabilistic sensitivity analysis (PSA) was conducted to account for uncertainty in model parameters. Monte Carlo simulations were performed, drawing values from specified probability distributions for each parameter (Table 1). Results are presented on a cost-effectiveness plane to visualise the joint distribution of cost and outcomes and the uncertainty in the ICER, and also as a cost-effectiveness acceptability curve (CEAC), showing the probability of cost-effectiveness across the range of thresholds. Confidence intervals for the difference in costs and in outcomes were taken from the distributions of the simulations; we did not attempt to put a confidence interval on the ICER as these are sensitive to the analytical method used but prefer the cost-effectiveness plane and the CEAC as a means to visualise the uncertainty in the estimated ICER and in decision-making.

##### Role of the funding source

No involvement in the study design, data collection, analysis and interpretation, writing of the report or the decision to submit for publication.

## Results

### Patient characteristics and survival

Of the 86 study patients, 66 (77%) were male, and 46 (53%) were from Tanzania. Over 80% had first-line treatment, and 33% received second-line (Additional File 6: Table 1). When stratified by disease stage, patients with limited-stage BL had a 24-month survival rate of 70% (95% CI, 48%–84%) compared to 45% (95% CI, 28%–60%) for advanced-stage BL (Additional File 6: Fig. 1).

### Base case analysis

**Fig. 2:**
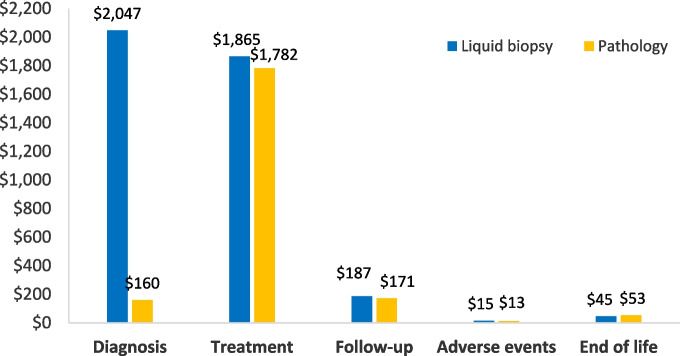
Mean costs per patient by cost category. Costs by type for each diagnosis approach in the model (shown in US $)

Figure 2 presents the mean costs per patient by cost category from the model. Liquid biopsy showed notably higher costs for diagnosis due to the higher cost per sample and the NNT multiplier. Treatment costs were slightly higher because limited-stage patients tend to survive longer during treatment, but all other costs were comparable between the two arms.

The base case analysis showed that the liquid biopsy arm was more costly but with improved health outcomes compared to histopathology (Table 2). The total cost per patient for liquid biopsy was TZS 5,132,899 ($1978) higher than for histopathology. Liquid biopsy resulted in a per-patient burden of 9.4 DALYs compared to 10.5 DALYs with histopathology, averting 1.1 DALYs per patient. This led to an ICER of TZS 4,614,371 ($1778) per DALY averted. This would be considered cost-effective at a 3 × GDP threshold, but not at the others considered.

**Table 2:** Cost-effectiveness of liquid biopsy compared to pathology: base case and scenario analyses

	**Liquid biopsy**		**Pathology**		**ICER**	**ICER**
	**Cost (TZS)**	**DALYs**	**Cost (TZS)**	**DALYs**	**(TZS)**	**($)**
**A Base case**	10,773,354	9.39	5,640,455	10.50	4,614,371	1778
**B Number needed to test**						
2	8,656,843	9.39	#	#	2,711,671	1045
3	10,215,487	9.39	#	#	4,112,860	1585
*4	10,773,354	9.39	#	#	4,614,371	1778
5	12,081,803	9.39	#	#	5,790,641	2231
6	13,390,253	9.39	#	#	6,966,911	2685
**C Availability of liquid biopsy**						
20%	7,349,250	10.28	#	#	7,680,861	2960
40%	8,106,185	10.06	#	#	5,541,603	2135
60%	9,339,050	9.83	#	#	5,541,603	2135
80%	10,159,345	9.61	#	#	5,077,988	1957
*100%	10,773,354	9.39	#	#	4,614,371	1778

A Base case analysis

B Varying the number-needed-to-test per BL case identified, to reflect the cost of tests done on patients diagnosed with cancers other than BL. Pathology costs and DALY burden, and liquid biopsy DALY burden, remain as the base case

C Varying the availability of the liquid biopsy diagnostic, by a weighted sum of the costs and outcomes for each arm, taking account of the increase in per-patient liquid biopsy cost with decreased throughput

ICERs shown in both Tanzanian Shillings (TZS) and US Dollars ($). Further details in Additional File 7

^*^Base case; # values as for the base case; *ICER*, incremental cost-effectiveness ratio, $ per DALY averted

### Sensitivity analysis

**Fig. 3:**
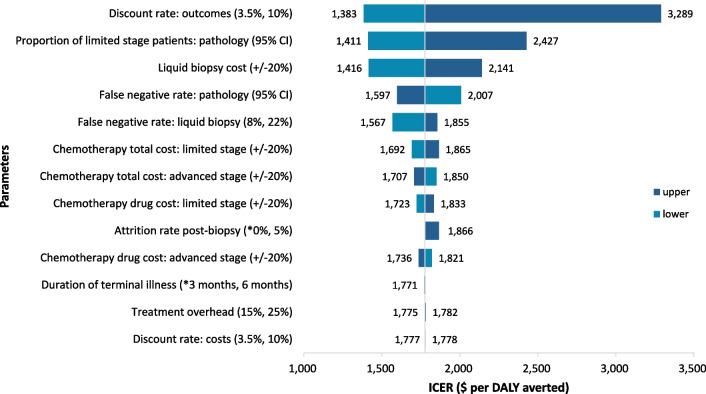
Results of one-way sensitivity analyses. *ICER*, incremental cost-effectiveness ratio; *DALY*, disability-adjusted life year. The asterisk “*” indicates the base case value

One-way sensitivity analysis (Fig. 3) showed that the ICER was sensitive to the discount rate for outcomes, but not for costs, as the costs after year 1 are similar for both arms. The ICER was sensitive to the cost of liquid biopsy diagnosis and the proportion of limited-stage patients at current presentation. However, at the ranges tested, none of the parameters resulted in an ICER above our highest threshold of 3 × GDP.

**Fig. 4:**
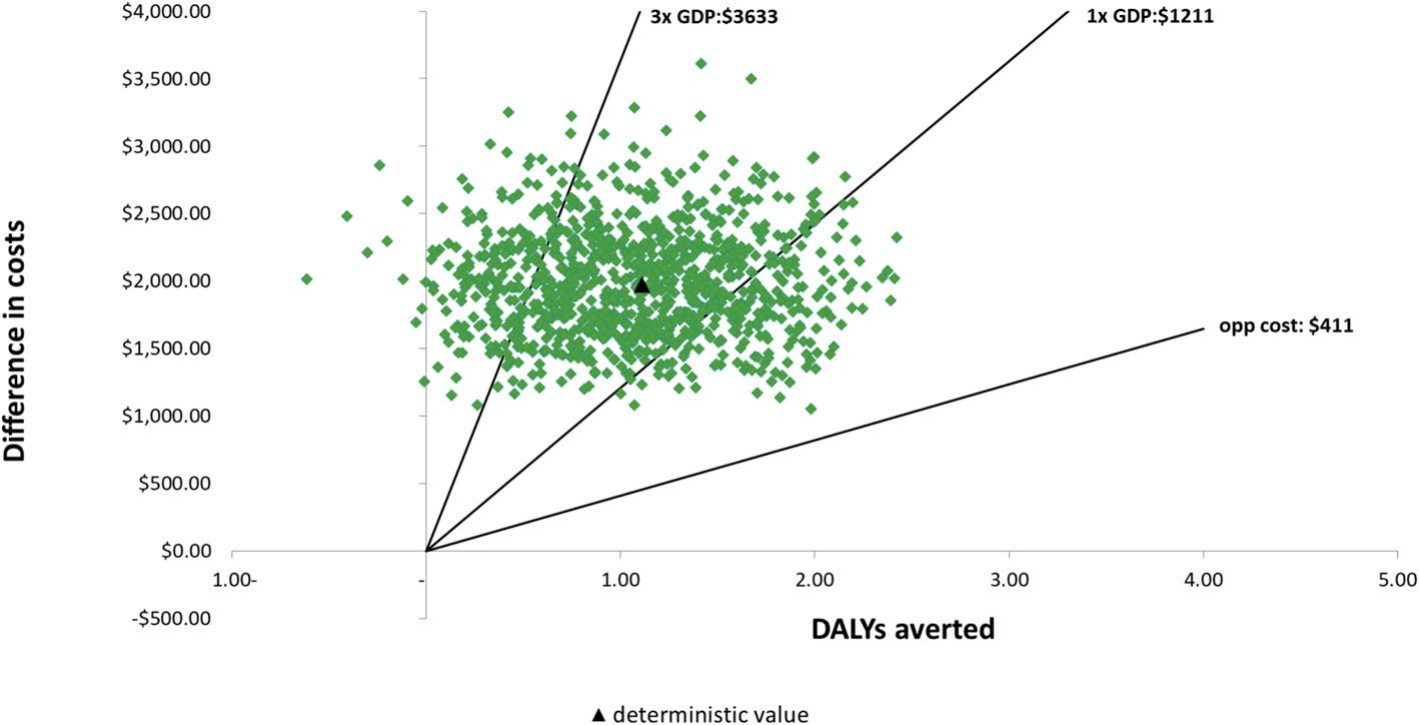
Cost effectiveness plane. Plot of incremental cost and DALYs averted for 1000 iterations in the probabilistic sensitivity analysis. The gradient of each line represents possible cost-effectiveness thresholds; points falling below and to the right of each line would be considered cost-effective. *DALY*, disability-adjusted life year

**Fig. 5:**
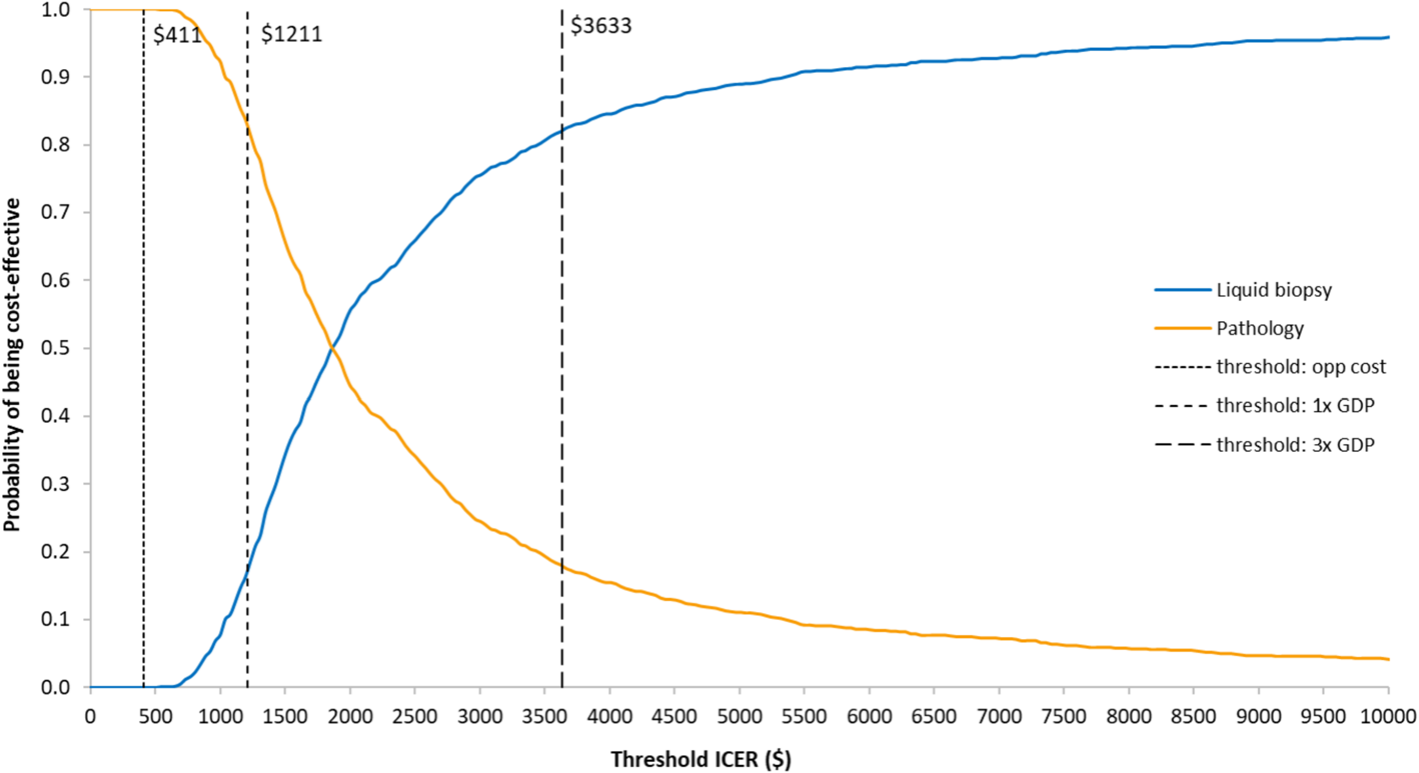
Cost effectiveness acceptability curve. Probability of each option being cost effective with increasing cost-effectiveness threshold. The three considered thresholds are shown as vertical bars. *ICER*, incremental cost-effectiveness ratio; *GDP*, gross domestic product; *opp cost*, opportunity cost

Results of PSA are visualised in Fig. 4, showing varying probabilities of being cost-effective for the illustrative thresholds. The PSA identifies 95% confidence intervals for the difference in costs ($1299–2840) and in outcomes (0.13–2.06 DALYs). The cost-effectiveness acceptability curves indicate that liquid biopsy was more likely to be cost-effective than histopathology for any willingness-to-pay above $1890 (Fig. 5).

**Fig. 6:**
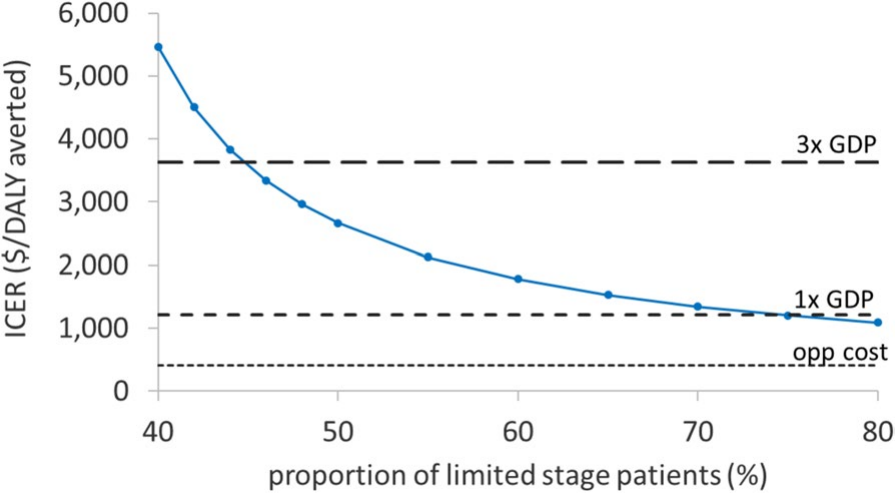
Scenario analysis—proportion of limited-stage patients at earlier diagnosis. Varying the proportion of limited stage patients at diagnosis by liquid biopsy, from 40% (the proportion in current presenting population) to 80% (i.e. assuming that half the patients who have limited-stage at first contact, would have advanced disease if they subsequently presented to a cancer centre, 3–4 weeks later). The three thresholds considered are shown as horizontal bars. *ICER*, incremental cost-effectiveness ratio; *DALY*, disability-adjusted life year; *GDP*, gross domestic product; *opp cost*, opportunity cost

The ICER is sensitive to the assumed shift in disease stage as a result of the earlier diagnosis. When the assumed proportion of limited-stage patients at first contact (intervention arm) was assumed to be the same as in the current presenting population (40%, i.e. assuming no stage shift), the ICER was above 3 × GDP, falling below this threshold with an assumption of 45% limited-stage patients in the first-contact population, and below 1 × GDP with 75% (Fig. 6).

When the number-needed-to-test multiplier was increased to 6 (i.e. only 1 in 6 of the tested patients is found to have BL), the ICER was higher but remained within the range of plausible cost-effectiveness thresholds (Table 2; Additional File 7: Table 1). Reducing the assumed availability of the liquid biopsy test from 100% in the base case led to higher ICERS (Table 2; Additional File 7: Table 2) due to higher per-patient costs of the liquid biopsy test at decreased throughput, and fewer DALYs averted given fewer patients had the opportunity of earlier diagnosis.

Extending the duration of disease risk beyond 2 years led to a decrease in the ICER, as patients in the control arm incurred greater increases in morbidity, mortality and mortality-related costs (Additional File 7: Table 3). Relaxing the assumption that FN patients have the prognosis of advanced stage patients led to an increased ICER of $2502 (Additional File 7: Table 4). Shortening the model cycle to 1 week for the first 2 years of the model made little difference to the ICER (Additional File 7: Table 4) in this DALY-based model, as the DALY burden is predominantly due to the estimated 65 lost life years; the small differences are largely due to higher estimates of follow-up costs and more precise discounting with the shorter model cycle. The scenario based only on Tanzanian patients had an ICER of $1193 but with increased uncertainty due to the smaller sample size (Additional File 7: Table 4); the difference is due to a larger number of DALYs averted than in the total sample, which likely relates to case-mix differences given our main Tanzanian study site is a large capital-city cancer centre, in contrast to the rural Ugandan site.

When rituximab costs and its effect on outcomes were excluded, the ICER was $1591 (Additional File 5: Fig. 2, Table 2).

## Discussion

This paper reports the first cost-effectiveness study examining liquid biopsy for childhood cancer detection in SSA using locally collected laboratory and patient data. The findings suggest that liquid biopsy can be a cost-effective diagnostic for BL in Tanzania depending on the cost-effectiveness threshold used by decision-makers, at an ICER of $1778/DALY averted in the base case. Scenario analysis found that the ICER is sensitive to the stage shift assumed due to the earlier diagnosis, rising above 3 × GDP if no stage shift is assumed. The ICER increases but remains below 3 × GDP if only one in six of the tested patients was found to have BL compared to the base case of one in four, or if liquid biopsy diagnosis is available to only 20% of incident cases. The findings are robust across the range of parameters tested in univariate sensitivity analysis.

The model’s improved health outcomes for the liquid biopsy diagnosis rely on earlier diagnosis, by making diagnosis accessible at first contact with health care. There is further benefit from the increased sensitivity compared to histopathology, reducing the number of patients who experience delays in accessing the right treatment following initial misdiagnosis. There may be additional benefits from reducing geographic inequities in access to diagnosis. In this cancer affecting predominantly children, with few long-term sequelae for patients who survive beyond 2 years, each life saved averts a substantial number of lost life years.

The difference in cost between histopathology and liquid biopsy is largely due to the higher cost of the liquid biopsy diagnosis. These costs are expected to reduce over time. The sequencing costing used in this analysis is based on technology available at the start of this study in 2019 [37]; newer technologies now offer the promise of high-throughput genome sequencing for under $100 [47]. Further, increased use of sequencing-based diagnostics in the region will allow greater use of higher capacity sequencing machines at lower cost per sample. Such expansion requires improved local supply chains for sequencing equipment and reagents, which will also improve costs [37]. Further, the NNT multiplier inflates the liquid biopsy costs; this is expected to reduce with further panel development to include mutation signatures of additional cancers, which would also contribute to cost reduction through increased throughput. These reductions in cost will improve both the cost-effectiveness and the affordability of sequencing-based diagnostics, including the BL case.

We found no comparable published cost-effectiveness analysis of targeted sequencing of patient DNA for diagnosis, in a sub-Saharan African setting, although treatment of BL has been shown to be cost-effective in Uganda [34] and Ghana [48], as has treatment of childhood cancers in general [35, 39]. A recent systematic review of economic analyses of next-generation sequencing in diagnosis noted that few were conducted in low- or middle-income countries [49]. A review of the use of one sequencing technology in Africa found the vast majority of applications were in disease surveillance rather than diagnosis; only 5 of their 93 studies included any costing, and none considered cost-effectiveness [50]. Further, comparisons of the economics of sequencing-based diagnostics are notoriously challenging, as costs vary widely over time, and with technology and sequencing parameters (such as depth of sequencing).

### Study limitations

We modelled a hypothetical intervention arm that made use of the benefits of liquid biopsy, with the blood sample taken at first contact. Whilst this could improve outcomes, we have not demonstrated the feasibility of this approach, which would require establishment of an efficient sample transport mechanism, and a prompt referral pathway directly to a cancer centre for diagnosed cases. There are examples of sample transport mechanisms which could be adopted; these include HIV viral load, HPV and Covid serology testing centralised at the National Reference Laboratory in Dar-es-Salaam. Beyond infectious disease, the Tanzanian paediatric oncology network transports tissue and blood samples for analysis at pathology facilities in Dar-es-Salaam. Models from neighbouring countries could also be informative, such as Uganda’s use of motorbike riders to deliver samples from rural areas to transport hubs. Rapid referral could follow the model already adopted for other diseases such as HIV testing [51]. However, the feasibility of this pathway revision has not been demonstrated and would require large-scale training of primary care healthcare professionals. A feasibility or pilot study is needed.

As an alternative or interim approach, can liquid biopsy be cost-effective at presentation to a cancer centre? The ICER would be higher than presented here, with little early-diagnosis benefit. However, the cost could be reduced via a combined pathway, using histopathology first to identify likely BL patients. The histopathology protocol for BL is a staged process [52]; the initial morphology step is relatively inexpensive [37], and experienced pathologists find they can detect up to 80% of BL patients at this step [52]. This would enrich the liquid biopsy-tested population for BL cases, bringing down the number tested per case and hence the cost. The cost per case would also be reduced by panels designed to detect more than one condition. Hence, a combination of these developments could improve the cost-effectiveness of liquid biopsy for these childhood cancers without involving local facilities. Further work is needed to determine a cost-effective combined pathway.

The model is limited in its reflection of the constraints of health care in a sub-Saharan African country. For example, we model a cohort of diagnosed BL patients and do not take into account those patients who do not present at a cancer centre or die before achieving a diagnosis [14]. Treatment abandonment is a known problem, with reports of up to 34% abandonment in some centres [11] for reasons of logistics, affordability and health beliefs and understanding [14]; our model does not explicitly allow for abandonment, other than in the resultant effect on survival. Further, we have not explicitly accounted for dose reduction and other treatment adjustments to accommodate toxicity or comorbidities, other than as reflected in cycle counts. Hence, the model presents an ‘ideal’ version of reality, and there may be missing costs or morbidity; they are, however, likely to have a similar effect on the two arms of the model.

A further limitation is the assumptions made in the design of the hypothetical intervention arm. Specifically, we assumed that with earlier diagnosis [19], there would be a higher proportion of limited-stage patients. We found that the ICER rose above 3 × GDP if there was no such shift in case mix; however, BL is an aggressive cancer, so it is likely that some disease progression would occur within a month in a child already sufficiently symptomatic to seek medical care. Other key assumptions—NNT per case identified by liquid biopsy and assuming advanced-stage outcomes for patients with FN diagnosis by pathology—affected the ICER, but it remained within the range of the considered thresholds.

Regarding generalisability, our cohort included patients from two East African countries, which enhances sample size and generalisability; however, costs and context are Tanzania-specific. The model can be adapted for settings with similar diagnostic access pathways by incorporating local costs and outcomes, but may not be applicable where diagnostic pathways differ.

In considering a cohort of *bona fide* diagnosed BL patients, we ignore the impact of false positive diagnoses. As the AI-REAL development work prioritised specificity in developing the diagnostic algorithm, the specificity is high [33]; we therefore expect a reduction in false positives and hence improved outcomes for these patients. In ignoring this improved outcome, our model is conservative.

Finally, the model assumes a perfect diagnostic yield, i.e. that all patients are diagnosed. This is typically not the case, due to sample quality or patient non-compliance. The diagnostic costing allows for an error rate at each step [37] but may not fully account for diagnostic failures. However, AI-REAL experience suggests these are likely to be higher for histopathology than liquid biopsy, so the effect on the ICER is conservative. For liquid biopsy, requirements for sample transport were determined by AI-REAL; incorporating these into routine practice will need focus in implementation or feasibility work.

### Policy implications

A key policy question from an economic perspective is delivery route, for example via a public/private not-for-profit enterprise, or incorporation into existing government laboratories. This may vary by country and will affect the implementation cost. In addition, there is a choice of whether to involve local health centres or focus (at least initially) on referral-hospital diagnosis using a combined histopathology/liquid biopsy protocol; a hospital approach is more readily implemented but has less impact on health outcomes, as fewer benefits of early diagnosis will be realised, nor any broadened access to diagnosis due to the more local biopsy.

Once the implementation route is defined, further work is needed to estimate overall budget impact, including implementation costs. One component would be start-up costs of sequencing technologies as a service, outside the academic laboratory where the AI-REAL study developed capability. The cost of equipment is already included in the per-patient cost estimate, so this implementation work includes laboratory start-up, procedure validation and quality certification. This cost is likely to be lower than that incurred during AI-REAL, given the local capability developed by the project, and may in fact not be borne directly by the health system, but by a third party. Alternatively, in Uganda, a government laboratory partnered with AI-REAL has also developed sequencing capability and would be able to absorb lymphoma samples with little additional investment; this route may be applicable in other countries. A second component of implementation cost would be creating awareness and technical expertise among health care professionals, whether in cancer centres or local facilities, to enable them to use the new diagnostic effectively. A feasibility study would allow estimation of such costs.

Finally, policy support for strengthening local biotechnology infrastructure would enable more sustainable costs and expand the use of this promising technology.

## Conclusions

The findings indicate that for BL in children and young adults in Tanzania, diagnosis by liquid biopsy has the potential to improve detection and early access to correct treatment, expected to increase survival rates in this aggressive childhood cancer. This diagnostic approach could be cost-effective, depending on the assumptions and thresholds used.

## Supplementary Information

Additional file 1: CHEERS checklist.

Additional file 2: survival modelling.

Additional file 3: age-related morbidity and mortality.

Additional file 4: unit costs and resource use.

Additional file 5: no-rituximab analysis.

Additional file 6: patient characteristics.

Additional file 7: scenario analyses.

## Data Availability

The anonymised data and model that support the findings of this study are available from the corresponding author on reasonable request.

## Abbreviations

BL: Burkitt lymphoma
SSA: Sub-Saharan Africa
DALY: Disability-Adjusted Life Year
ICER: Incremental cost-effectiveness ratio
GDP: Gross domestic product
LMIC: Low- and middle-income country
HIC: High-income country
ctDNA: Circulating tumour DNA
AI-REAL: Aggressive Infection-Related East Africa Lymphoma
CHEERS: Consolidated Health Economics Reporting Standard
FN: False negative
NNT: Number needed to test
GBD: Global Burden of Disease
YLL: Years of life lost
YLD: Years lived with disability
PSA: Probabilistic sensitivity analysis
CI: Confidence interval
TZS: Tanzanian shillings
