# Supplementary material for: Is liquid biopsy a cost-effective method to diagnose Burkitt Lymphoma in children and young adults? A health economic evaluation in Tanzania
Source: BMC Med. 2026 Feb 21;24:180. doi: 10.1186/s12916-026-04694-2 (PMC13032632; doi:10.1186/s12916-026-04694-2)
Supplement: Supplementary file 2 — Additional file 2: survival modelling. [file 12916_2026_4694_MOESM2_ESM.pdf]

ADDITIONAL FILE 2:

Table 1 Alternative survival models

Model fit statistics, and resultant ICER

Model	Observations	Degrees of freedom	AIC	BIC	ICER
Weibull	85	3	226	233	$1,778
exponential	85	2	250	255	$1,379
Gompertz	85	3	230	237	$1,896
lognormal	85	3	226	234	$2,269
loglogistic	85	3	225	232	$1,873

AIC Akaike Information Criterion; BIC Bayesian Information Criterion; ICER Incremental Cost Effectiveness Ratio (dollars per DALY averted)

AIC and BIC assess the fit of the model whilst accounting for the number of parameters, to avoid over-fitting. Lower values indicate improved fit. BIC penalises additional parameters more strongly than AIC.

Table 2 Matrix of transition probabilities between health states in the Markov model

	TO:			
FROM:	On treatment	Post treatment	Cured	Dead
On treatment		1-p(w)		p(w)
Post-treatment		1-p(w)		p(w)
Post treatment (2 years)			1-p(w)	p(w)
Cured			1-p(m)	p(m)
Dead				1

Time in model (cycles)	1	2	3	4	5	6	7	8
State	On treatment	Post treatment						
p(w) limited stage patients	0.110	0.047	0.036	0.031	0.027	0.025	0.023	0.021
p(w) advanced stage patients	0.260	0.117	0.091	0.077	0.069	0.062	0.057	0.054

p(w) time-dependent probability of death estimated from the Weibull model

p(m) age-dependent mortality

Shaded boxes represent non-allowed transitions. After 2 years, patients experience population age-dependent rates of mortality, shown in Additional File 3.
