# Supplementary material for: Is liquid biopsy a cost-effective method to diagnose Burkitt Lymphoma in children and young adults? A health economic evaluation in Tanzania
Source: BMC Med. 2026 Feb 21;24:180. doi: 10.1186/s12916-026-04694-2 (PMC13032632; doi:10.1186/s12916-026-04694-2)
Supplement: Supplementary file 3 — Additional file 3: age-related morbidity and mortality. [file 12916_2026_4694_MOESM3_ESM.pdf]

ADDITIONAL FILE 3:

Table 1 Age-related model inputs taken from the Global Burden of Disease study, 2019

Age band	Life expectancy (years)	Survival probability (quarterly)	DALY weighting
10-14 years	61.40	0.9998	0.064
15-19 years	56.60	0.9997	
20-24 years	51.90	0.9996	0.082
25-29 years	47.28	0.9995	0.098
30-34 years	42.74	0.9993	0.112
35-39 years	38.28	0.9991	0.122
40-44 years	33.94	0.9987	0.135
45-49 years	29.75	0.9983	0.146
50-54 years	25.70	0.9976	0.158
55-59 years	21.84	0.9967	0.172
60-64 years	18.16	0.9950	0.187
65-69 years	14.80	0.9926	0.209
70-74 years	11.73	0.9885	0.236
75-79 years	9.12	0.9826	0.262
80-84	6.90	0.9732	0.304
85-89	5.16	0.9629	
90-94		0.9480	
95+ years		0.9239	

Source:

https://ghdx.healthdata.org/gbd-2019
