# Supplementary material for: Is liquid biopsy a cost-effective method to diagnose Burkitt Lymphoma in children and young adults? A health economic evaluation in Tanzania
Source: BMC Med. 2026 Feb 21;24:180. doi: 10.1186/s12916-026-04694-2 (PMC13032632; doi:10.1186/s12916-026-04694-2)
Supplement: Supplementary file 4 — Additional file 4: unit costs and resource use. [file 12916_2026_4694_MOESM4_ESM.pdf]

ADDITIONAL FILE 4:

ADDITIONAL FILE 4 Table 1: Chemotherapy drugs unit costs and resource use

	**Unit costs (TZS): chemotherapy drug**				**Resource use: drug dosing per cycle** Regimen:							
					COM	R-COM	COP	CHOP	R-CHOP	EMIC/ IVAC$^+$	R-EMIC/ R-IVAC$^+$	Triple IT
**Agent**	Price (TZS)	Size	Unit	Unit cost (mg or ml)	mg/m^2	mg/m^2	mg/m^2	mg/m^2	mg/m^2	mg/m^2	mg/m^2	mg/m^2
**Dosed in mg/m^2:**												
Cyclophosphamide	14,500	1000	mg	15	1200	1200	300	750	750			
Vincristine	6,400	1	mg	6400	1.4	1.4	1	1	1			
Methotrexate	25,000	50	mg	500	75	75						
Doxorubicin	68,650	50	mg	1373				50	50			
Prednisolone	15,560	1400	mg	11			420	500	500			
Etoposide	15,600	100	mg	156						180	180	
Cytarabine	15,600	100	mg	156						300	300	
Ifosfamide	48,100	1000	mg	48						4500	4500	
Mesna	4,550	200	mg	23	350	350				5400	5400	
Dextrose + Normal Saline	6,400	1	litre	6	500	500	500	500	500	9000	9000	
**Dosed in mg:**					mg	mg	mg	mg	mg	mg	mg	mg
Rituximab	195,478	100	mg	1955		300*			300*		300*	
IT hydrocortisone	8,760	100	mg	88								50
IT methotrexate	25,000	50	mg	500	24	24	12	#	#	24	24	12
IT cytarabine	15,600	100	mg	156	50	50	50	#	#	50	50	50

Agent	Drug cost per cycle (TZS)<br>Regimen:<br>COM	R-COM	COP	CHOP	R-CHOP	EMIC/<br>IVAC $^{+}$	R-EMIC/<br>R-IVAC $^{+}$	Triple IT
	TZS/m^2	TZS/m^2	TZS/m^2	TZS/m^2	TZS/m^2	TZS/m^2	TZS/m^2	TZS/m^2
Dosed in mg/m2:								
Cyclophosphamide	17,400	17,400	4,350	10,875	10,875			
Vincristine	8,960	8,960	6,400	6,400	6,400			
Methotrexate	37,500	37,500						
Doxorubicin				68,650	68,650			
Prednisolone			4,668	5,557	5,557			
Etoposide						28,080	28,080	
Cytarabine						46,800	46,800	
Ifosfamide						216,450	216,450	
Mesna	7,963	7,963				122,850	122,850	
Dextrose + Normal Saline	3,200	3,200	3,200	3,200	3,200	57,600	57,600	
	TZS	TZS	TZS	TZS	TZS	TZS	TZS	TZS
Dosed in mg:								
Rituximab		586,434			586,434		586,434	
IT hydrocortisone								4,380
IT methotrexate	12,000	12,000	6,000	#	#	12,000	12,000	6,000
IT cytarabine	7,800	7,800	7,800	#	#	7,800	7,800	7,800

Notes:

+ EMIC and IVAC are alternative abbreviations for the same regimen

* rituximab dosage per-protocol is 375mg/m^2; in practice given 100mg vials and typical patient BSA of ~1m^2, dosage was typically 3 complete vials

IT treatment is on a case-by-case basis with CHOP, for rare extra-nodal sites. In absence of complete documentation, we assume no IT treatment for the small number of study patients who were treated with CHOP or R-CHOP

IT intrathecal dosing; TZS Tanzanian Shillings

Total cost per patient is derived by multiplying the sub-total per-cycle cost for drugs dosed per m^2 by the patient's body surface area (BSA), and adding the sub-total cost for drugs dosed per mg

Patient BSA is derived from the patient's weight in kg, using the formula of Furqan and Haque, 2009 [1] which approximates to the Dubois formula based on height and weight

1. Furqan M, Haque A. Surface area in children: a simple formula. Indian Pediatr. 2009 Dec;46(12):1085-7. Epub 2009 Apr 1. PMID: 19430073.

ADDITIONAL FILE 4 Table 2: Chemotherapy non-drug unit costs and resource use

**First line (COM)**		**Unit cost (TZS)**	**Resource use**							
			Investigations and staging	**COM** Cycle 1	Cycle 2	Cycle 3	Cycle 4	Cycle 5	Cycle 6	**COP cycle**
Laboratory	Complete blood count	10,000	1	1	1	1	1	1	1	
	Lactate dehydrogenase	10,000		2						
	Creatinine	6,000	1	1	1	1	1	1	1	
	Bilirubin	12,000	1	1	1	1	1	1	1	
	Thromboplastin	35,000	1	1						
	Uric acid, Potassium, Calcium, Phosphate [1]	40,000 [3]		3						
Imaging and staging	Abdominal ultrasound	15,000	1		1					
	Chest X-ray	20,000	1		1					
	CT scan	350,000	1						1	
	Lumbar puncture [2]	5,000	1	3 [4]	3	3	3	3	3	
	Bone marrow biopsy	80,000	1							
	CSF cytology	30,000	1							
Chemo delivery and support costs	Additional fluids - litres	4,000		8						
	Chemo administration	10,000		1	1	1	1	1	1	1
	Anti-vomiting medication (Emeset)	635		1	1	1	1	1	1	1
Visits	Index consultation	25000	1							
	In-patient days	10,000		5						
	Outpatient clinic visit	5000	1	1	1	1	1	1	1	1
	New patient file	30000	1							

**First line (COM)**	(continued)	**Cost per cycle** Investigations and staging	**COM** Cycle 1	Cycle 2	Cycle 3	Cycle 4	Cycle 5	Cycle 6	**COP cycle**
Laboratory	Complete blood count	10,000	10,000	10,000	10,000	10,000	10,000	10,000	
	Lactate dehydrogenase		20,000						
	Creatinine	6,000	6,000	6,000	6,000	6,000	6,000	6,000	
	Bilirubin	12,000	12,000	12,000	12,000	12,000	12,000	12,000	
	Thromboplastin	35,000	35,000						
	Uric acid, Potassium, Calcium, Phosphate [1]		120,000						
Imaging and staging	Abdominal ultrasound	15,000		15,000					
	Chest X-ray	20,000		20,000					
	CT scan	350,000						350,000	
	Lumbar puncture [2]	5,000	15,000	15,000	15,000	15,000	15,000	15,000	
	Bone marrow biopsy	80,000							
	CSF cytology	30,000							
Chemo delivery and support costs	Additional fluids - litres		32,000						
	Chemo administration		10,000	10,000	10,000	10,000	10,000	10,000	10,000
	Anti-vomiting medication (Emeset)		635	635	635	635	635	635	635
Visits	Index consultation	25,000							
	In-patient days		50,000						
	Outpatient clinic visit	5,000	5,000	5,000	5,000	5,000	5,000	5,000	5,000
	New patient file	30,000							
**TOTAL**		**623,000**	**315,635**	**93,635**	**58,635**	**58,635**	**58,635**	**408,635**	**15,635**

**Second line (EMIC/IVAC)**	**IF SWITCHING**	**Unit cost (TZS)**	**Resource use**				
			Investigations before starting 2nd line [5]	Cycle 1	Cycle 2	Cycle 3	Cycle 4
Laboratory	Complete blood count	10,000		1	1	1	1
	Lactate dehydrogenase	10,000		2			
	Creatinine	6,000		1	1	1	1
	Bilirubin	12,000		1	1	1	1
	Thromboplastin	35,000					
	Uric acid, Potassium, Calcium, Phosphate	40,000 [3]					
Imaging and staging	Abdominal ultrasound	15,000					
	Chest X-ray	20,000					
	CT scan	350,000					1
	Lumbar puncture [2]	5,000		3 [4]	3	3	3
	Bone marrow biopsy	80,000					
	CSF cytology	30,000					
Chemo delivery and support costs	Additional fluids - litres	4,000					
	Chemo administration	10,000		1	1	1	1
	Anti-vomiting medication (Emeset)	635		1	1	1	1
Visits	Index consultation	25000					
	In-patient days	10,000		5	5	5	5
	Outpatient clinic visit	5000	1	1	1	1	1

**<u>Second line (EMIC/IVAC)</u>**	**IF SWITCHING** (continued)	**Cost per cycle**				
		Investigations before starting 2nd line	Cycle 1	Cycle 2	Cycle 3	Cycle 4
Laboratory	Complete blood count		10,000	10,000	10,000	10,000
	Lactate dehydrogenase		20,000	0	0	0
	Creatinine		6,000	6,000	6,000	6,000
	Bilirubin		12,000	12,000	12,000	12,000
	Thromboplastin					
	Uric acid, Potassium, Calcium, Phosphate					
Imaging and staging	Abdominal ultrasound					
	Chest X-ray					
	CT scan					350,000
	Lumbar puncture		15,000	15,000	15,000	15,000
	Bone marrow biopsy					
	CSF cytology					
Chemo delivery and support costs	Additional fluids - litres					
	Chemo administration		10,000	10,000	10,000	10,000
	Anti-vomiting medication (Emeset)		635	635	635	635
Visits	Index consultation					
	In-patient days		50,000	50,000	50,000	50,000
	Outpatient clinic visit	5,000	5,000	5,000	5,000	5,000
**TOTAL**		**5,000**	**128,635**	**108,635**	**108,635**	**458,635**

**<u>Second line (EMIC/IVAC)</u>**	**IF RELAPSED**	**Unit cost (TZS)**	**Resource use**<br>Investigations before starting 2nd line [6]	Cycle 1	Cycle 2	Cycle 3	Cycle 4
Laboratory	Complete blood count	10,000	1	1	1	1	1
	Lactate dehydrogenase	10,000	1	2			
	Creatinine	6,000	1	1	1	1	1
	Bilirubin	12,000	1	1	1	1	1
	Thromboplastin	35,000					
	Uric acid, Potassium, Calcium, Phosphate	40,000 [3]		[7]			
Imaging and staging	Abdominal ultrasound	15,000	1				
	Chest X-ray	20,000	1				
	CT scan	350,000					1
	Lumbar puncture [2]	5,000	1	3 [4]	3	3	3
	Bone marrow biopsy	80,000	1				
	CSF cytology	30,000	1				
Chemo delivery and support costs	Additional fluids - litres	4,000		[7]			
	Chemo administration	10,000		1	1	1	1
	Anti-vomiting medication (Emeset)	635		1	1	1	1
Visits	Index consultation	25000	1				
	In-patient days	10,000		5	5	5	5
	Outpatient clinic visit	5000	1	1	1	1	1

**Second line (EMIC/IVAC)**	**IF RELAPSED** (continued)	**Cost per cycle**				
		Investigations before starting 2nd line	Cycle 1	Cycle 2	Cycle 3	Cycle 4
Laboratory	Complete blood count	10,000	10,000	10,000	10,000	10,000
	Lactate dehydrogenase	10,000	20,000	0	0	0
	Creatinine	6,000	6,000	6,000	6,000	6,000
	Bilirubin	12,000	12,000	12,000	12,000	12,000
	Thromboplastin					
	Uric acid, Potassium, Calcium, Phosphate					
Imaging and staging	Abdominal ultrasound	15,000				
	Chest X-ray	20,000				
	CT scan					350,000
	Lumbar puncture	5,000	15,000	15,000	15,000	15,000
	Bone marrow biopsy	80,000				
	CSF cytology	30,000				
Chemo delivery and support costs	Additional fluids - litres					
	Chemo administration		10,000	10,000	10,000	10,000
	Anti-vomiting medication (Emeset)		635	635	635	635
Visits	Index consultation	25,000				
	In-patient days		50,000	50,000	50,000	50,000
	Outpatient clinic visit	5,000	5,000	5,000	5,000	5,000
**TOTAL**		**218,000**	**128,635**	**108,635**	**108,635**	**458,635**

Notes:
1. Monitoring tumour lysis during first cycle
2. To take CSF sample, and for delivery of intrathecal chemotherapy
3. 10,000 for each measure
4. Assuming intrathecal chemotherapy at each cycle
5. Investigations are carried out at cycles 2 or 6 of first-line treatment to inform the decision to switch to second-line
6. Patients who relapse undergo investigations at relapse to inform the decision to start 2nd line treatment
7. Second-line patients assumed not to be at risk of tumour lysis syndrome

Calculating per patient cost:
All patients incur the initial diagnosis and staging cost
Patients incur the cumulative cost according to the number of cycles given
Patients with more than 6 cycles of COM incur costs as for a second round of cycles; cycle 7 = staging plus cycle 1, cycle 8 = cycle 2 and so on. This allows for additional investigations ahead of additional treatment.
Patients who switch directly from first to second line do so on the basis of evaluations at cycles 2 or 6 of first-line treatment, so these investigations are not repeated
Patients who relapse post-first-line treatment undergo investigations to assess the relapse and determine course of treatment; these investigations are assumed to replicate those at initial diagnosis
Patients with more than 4 cycles of EMIC/IVAC incur the cost of cycle 2 for each additional cycle

Abbreviations:
CSF - cerebrospinal fluid
COM - standard first-line chemotherapy of cyclophosphamide, vincristine and methotrexate, with intrathecal methotrexate and cytarabine
COP - standard prephase treatment of cyclophosphamide, vincristine and prednisolone
EMIC/IVAC - alternative abbreviations for standard second-line chemotherapy of etoposide, cytarabine and ifosfamide, with intrathecal methotrexate and cytarabine

ADDITIONAL FILE 4 Table 3: adverse event treatment unit costs and resource use

Toxicity	Measure	Treatment	Unit cost (TZS) [1]				
			Bednights	Medication [2]	Fluids	Tests	Consumables
Febrile neutropenia	(CTCAE grade 3-4)	IV antibiotics; fluids; blood cultures	10,000	20,000	4,000	25,000	10,000
Nausea	(CTCAE grade 3-4)	Fluids	10,000		4,000		10,000
Vomiting	(CTCAE grade 3-4)	Fluids	10,000		4,000		10,000
Diarrhoea	(CTCAE grade 3-4)	Fluids	10,000		4,000		10,000
Neutropenic sepsis	(CTCAE grade 3-4)	IV antibiotics; fluids; blood cultures	10,000	20,000	4,000	25,000	10,000

Toxicity	Resource use [3]						
	Bednights	Medication	(amount)	Fluids (litres per day)	Fluids (days)	Tests	Consumables
Febrile neutropenia	5	antibiotics (days)	5	3	3	2	1
Nausea	1			3	1		1
Vomiting	1			3	1		1
Diarrhoea	1			3	1		1
Neutropenic sepsis	5	antibiotics (days)	5	3	3	2	1

Toxicity	Total cost per episode	Frequency of AEs [4]		Number of episodes [5]		Average cost per patient	
		limited stage	advanced stage	limited stage	advanced stage	**limited stage**	**advanced stage**
Febrile neutropenia	246,000	1	2	5	2	**1,230,000**	**246,000**
Nausea	32,000	1	0	1	0	**32,000**	
Vomiting	32,000	0	1	0	4		**128,000**
Diarrhoea	32,000	0	1	0	7		**224,000**
Neutropenic sepsis	246,000	1	1	1	1	**246,000**	**246,000**

1 costs from AIREAL study hospital
2 antibiotic costed as piptazo, 100mg/kg
3 resource use data estimated by AIREAL clinicians
4 frequency counts from AIREAL data
5 some patients experienced multiple episodes of adverse events, resulting in an increase in the average cost per patient

ADDITIONAL FILE 4 Table 4: end-of-life care unit costs and resource use

**Care**		**Unit costs (TZS)** Price (TZS)	Size	Unit	Unit cost (TZS)
Palliative chemotherapy	Etoposide	15,600	100	mg	156
	Cyclophosphamide	14,500	1000	mg	15
	CHOP regimen: chemotherapy	94,682	1	per cycle per m^2	94,682
Pain relief	Oral morphine	4,407	56x10	mg	8
In-patient care	CHOP regimen: bednights	10,000	1	cycle	10,000
	End of life: bednights	10,000	1	cycle	10,000

**Care**		**Resource use** Probability	Source	Dosage	Frequency	Duration
Palliative chemotherapy	Etoposide	Used in 8 of 33 terminal patients	AIREAL	40mg per m^2	Daily	3 months [1]
	Cyclophosphamide	Assumed to be used in dual therapy for 67% of patients treated with etoposide	Clinical input	1200mg/m^2	3-weekly	3 months [1]
	CHOP regimen: chemotherapy	Used in 3 of 33 terminal patients	AIREAL	[2]	3-weekly	AIREAL [3]
Pain relief	Oral morphine	All terminal patients	Clinical input	0.5mg/kg	4-hourly	7 days
In-patient care	CHOP regimen: bednights	All CHOP patients	Protocol			21 days
	End of life: bednights	All terminal patients	Clinical input			7 days

Notes:

1 From AIREAL data, we find a mean duration of palliative chemotherapy of 3 months for oral etoposide
2 See Additional File 3a: chemotherapy unit cost and resource use, for details of CHOP chemotherapy regimen dosing
3 Cycle counts from AIREAL data

Using patient-level treatment data from AIREAL: palliative chemotherapy costs were applied to all patients who had reported palliative chemotherapy. Pain relief and end-of-life in-patient care were applied to all patients who died.
Total costs were divided by the number of patients who died (n=33); this per-patient cost was incurred for each death in the model.
