# Supplementary material for: Is liquid biopsy a cost-effective method to diagnose Burkitt Lymphoma in children and young adults? A health economic evaluation in Tanzania
Source: BMC Med. 2026 Feb 21;24:180. doi: 10.1186/s12916-026-04694-2 (PMC13032632; doi:10.1186/s12916-026-04694-2)
Supplement: Supplementary file 5 — Additional file 5: no-rituximab analysis. [file 12916_2026_4694_MOESM5_ESM.pdf]

ADDITIONAL FILE 5: NO-RITUXIMAB ANALYSIS

Figure 1: Overall survival for Burkitt lymphoma patients by use of rituximab at first-line

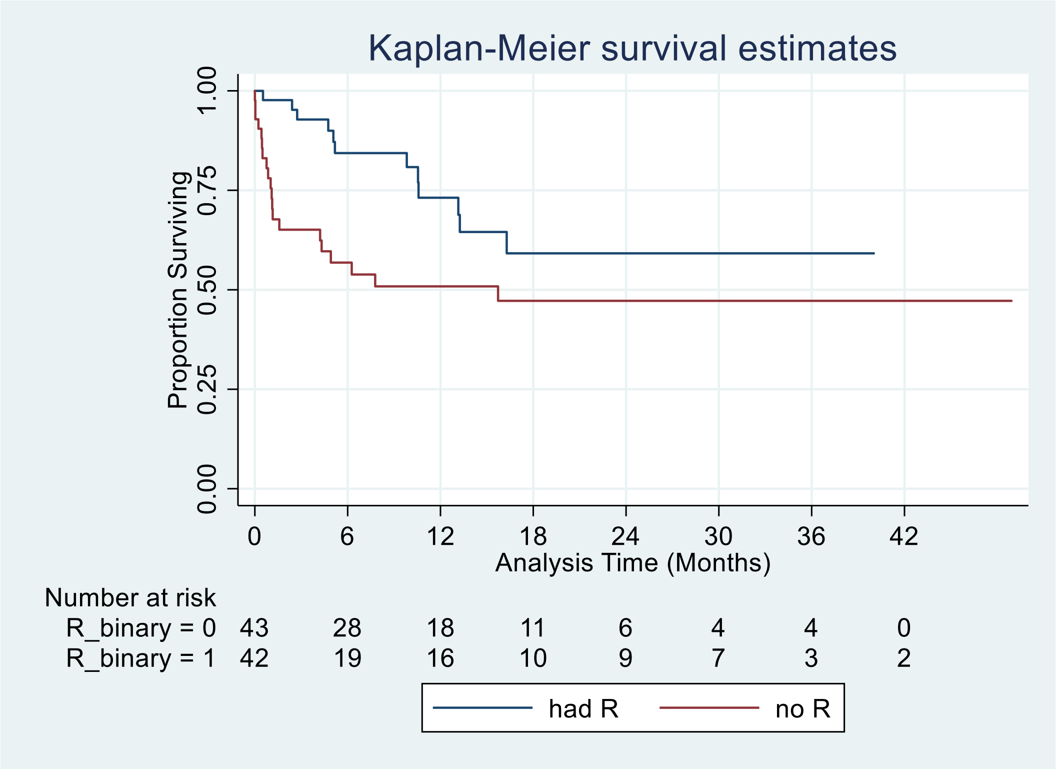

Table 1 Model input parameters for no-rituximab analysis

Parameter	Base case		No-rituximab scenario	
	mean	standard error	mean	standard error
Chemotherapy drug cost (TZS):				
-limited stage (n=36)	2,777,813	361,404	932,271	163,513
-advanced stage (n=52)	2,146,124	300,775	939,423	134,053
Weibull coefficients:				
-constant	-2.70	0.419	-3.06	0.475
-coefficient: advanced stage	0.95	0.410	0.91	0.412
-coefficient: no rituximab			0.70	0.366
-gamma	0.50	0.078	0.50	0.077
Calculated survival parameters:				
-lambda: limited stage	0.067		0.094	
-lambda: advanced stage	0.176		0.233	

Table shows parameters that are changed for the no-rituximab analysis; other parameters remain as in the base case

Table 2: cost-effectiveness of liquid biopsy compared to pathology when rituximab costs and effects are excluded

Intervention	Total costs TZS ($)	DALYs	Incremental costs TZS ($)	DALYs averted	Cost per DALY averted, ICER TZS ($)
**Liquid Biopsy**	8,877,882 (3421)	11.02	4,954,875 (1909)	1.20	4,128,895 (1591)
**Pathology**	3,923,006 (1512)	12.22			

Figure 2: cost-effectiveness acceptability curve when rituximab costs and effects are excluded

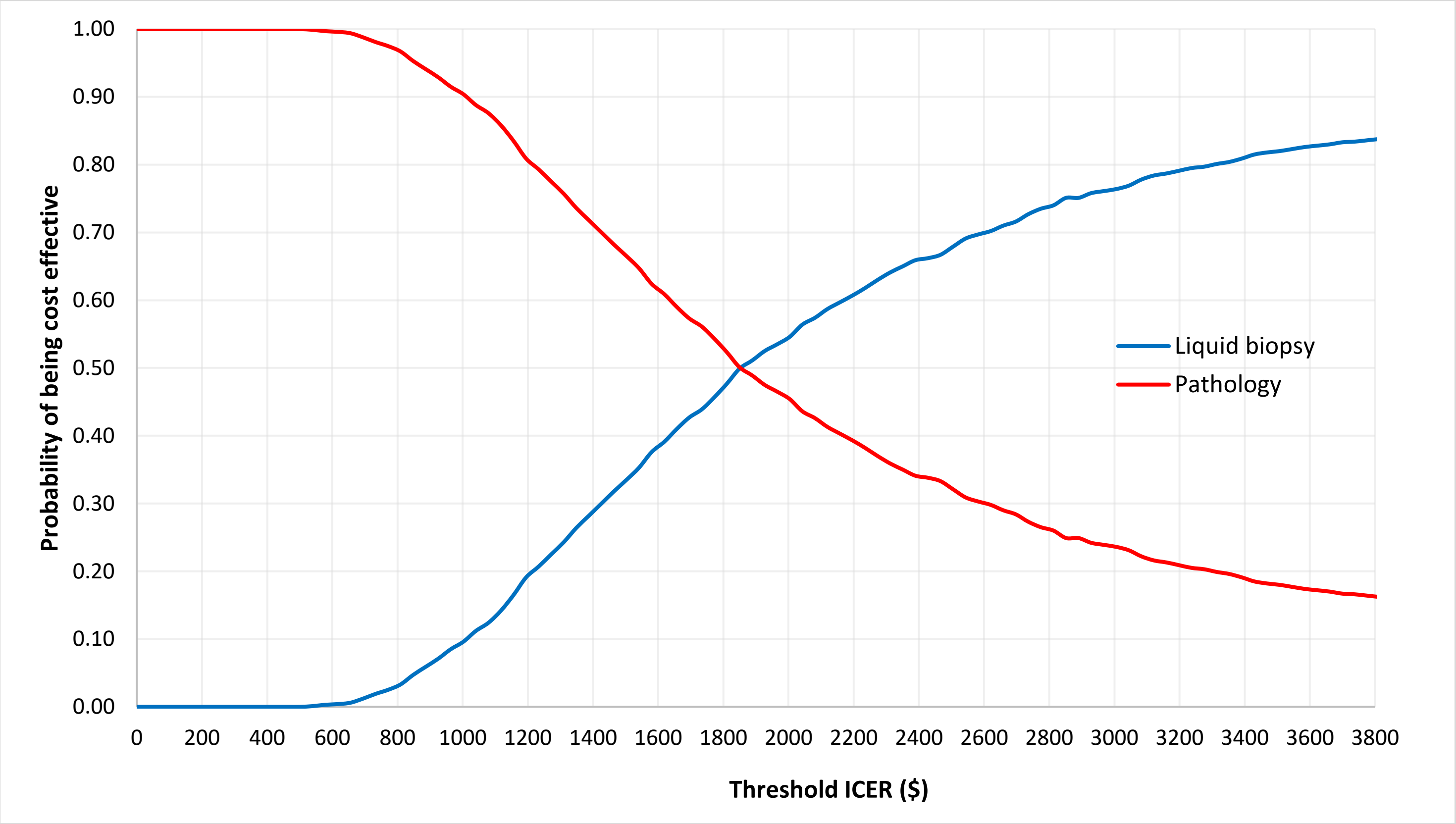

Probability of each alternative being cost effective, at a range of cost-effectiveness thresholds for determining whether the intervention of interest (diagnosis by liquid biopsy) is cost-effective. The commonly used threshold of 3-times GDP, is $3363.
