# Supplementary material for: Is liquid biopsy a cost-effective method to diagnose Burkitt Lymphoma in children and young adults? A health economic evaluation in Tanzania
Source: BMC Med. 2026 Feb 21;24:180. doi: 10.1186/s12916-026-04694-2 (PMC13032632; doi:10.1186/s12916-026-04694-2)

ADDITIONAL FILE 6:

Table 1 Patient characteristics and survival by disease stage

	**All BL patients**	**BL patients by disease stage:**	
		**Limited**	**Advanced**
	n=86	n=34	n=52
Male	66 (77%)	31 (91%)	35 (67%)
Age (years):			
Median	9	9	9
Mean	9.94	9.32	10.35
Country:			
Tanzania	46 (53%)	15 (44%)	31 (60%)
Uganda	40 (47%)	19 (56%)	21 (40%)
Treatment:			
Pre-phase: COP	9 (10%)	1 (3%)	8 (15%)
1st line: COM	75 (87%)	32 (94%)	43 (83%)
2nd line: EMIC	28 (33%)	12 (35%)	16 (31%)
Rituximab with 1st line	43 (50%)	22 (65%)	21 (40%)

One patient was excluded due to missing data on all treatment and survival measures

Chemotherapy regimens: COP – cyclophosphamide, vincristine, prednisolone. COM - cyclophosphamide, vincristine, methotrexate with intrathecal methotrexate and cytarabine. EMIC (also known as IVAC) – ifosfamide, etoposide, cytarabine

ADDITIONAL FILE 6:

Figure 1 Survival by disease stage

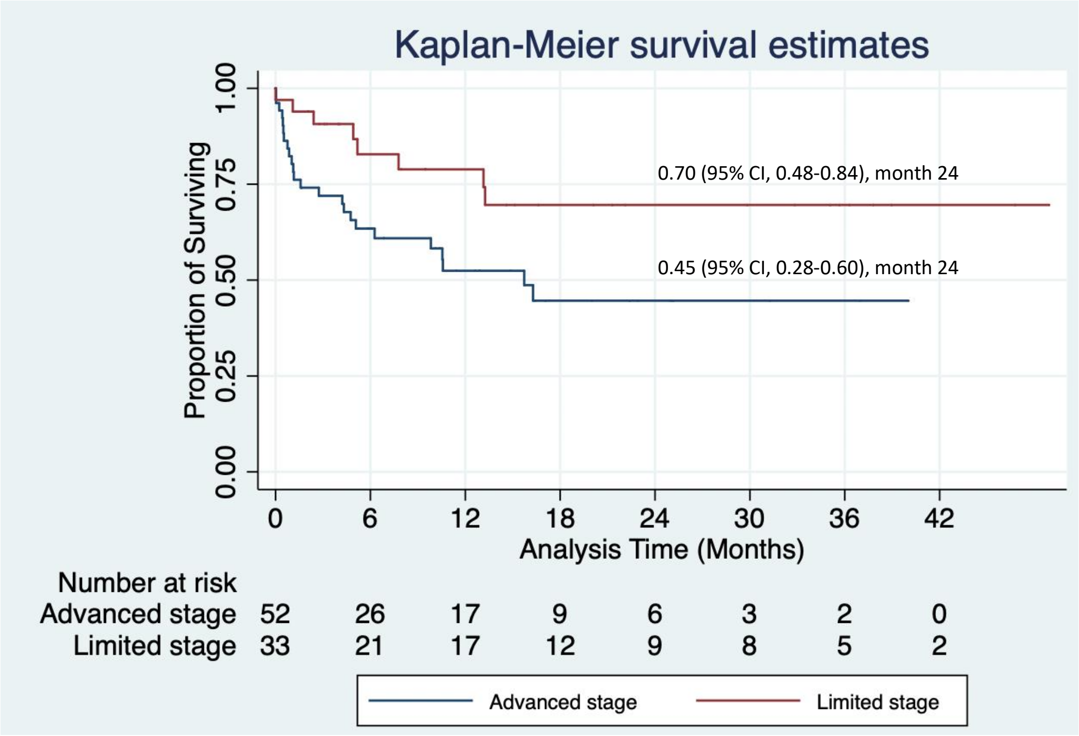

Supplement: Supplementary file 6 — Additional file 6: patient characteristics. [file 12916_2026_4694_MOESM6_ESM.pdf]
