# Supplementary material for: Is liquid biopsy a cost-effective method to diagnose Burkitt Lymphoma in children and young adults? A health economic evaluation in Tanzania
Source: BMC Med. 2026 Feb 21;24:180. doi: 10.1186/s12916-026-04694-2 (PMC13032632; doi:10.1186/s12916-026-04694-2)
Supplement: Supplementary file 7 — Additional file 7: scenario analyses. [file 12916_2026_4694_MOESM7_ESM.pdf]

ADDITIONAL FILE 7:

Table 1 Scenario analysis: varying number-needed-to-test per BL case identified

**Number needed to test**	**Liquid biopsy**		**Pathology**		**Incremental cost (TZS)**	**DALYs averted**	**ICER TZS ($)**
	Total cost (TZS)	Total DALYs	Total cost (TZS)	Total DALYs			
2	8,656,843	9.39	5,640,455	10.50	3,016,388	1.11	2,711,671 (1045)
3	10,215,487	9.39	5,640,455	10.50	4,575,032	1.11	4,112,860 (1585)
4*	10,773,354	9.39	5,640,455	10.50	5,132,899	1.11	4,614,371 (1778)
5	12,081,803	9.39	5,640,455	10.50	6,441,349	1.11	5,790,641 (2231)
6	13,390,253	9.39	5,640,455	10.50	7,749,798	1.11	6,966,911 (2685)

This table provides the complete results from varying the number-needed-to-test (NNT) between 2 and 6. The table presented in the main paper is a simplified version showing the columns that vary, i.e. liquid biopsy cost and the ICER.

TZS Tanzanian Shillings; $ US dollars; DALY disability-adjusted life year; ICER incremental cost effectiveness ratio, $ per DALY averted

* base case

ADDITIONAL FILE 7:

Table 2 Scenario analysis: varying the availability of the liquid biopsy diagnostic

Percentage availability	Estimated no. of samples/year	Liquid biopsy cost per patient (TZS)	Liquid biopsy		Pathology		Incremental cost (TZS)	DALYs averted	ICER TZS ($)
			Total cost (TZS)	Total DALYs	Total cost (TZS)	Total DALYs			
20	160	2,161,219	7,349,250	10.28	5,640,455	10.50	1,708,795	0.22	7,680,861 (2960)
40	320	1,556,306	8,106,185	10.06	5,640,455	10.50	2,465,730	0.44	5,541,603 (2135)
60	480	1,556,306	9,339,050	9.83	5,640,455	10.50	3,698,595	0.67	5,541,603 (2135)
80	640	1,437,378	10,159,345	9.61	5,640,455	10.50	4,518,890	0.89	5,077,988 (1957)
100*	800	1,308,450	10,773,354	9.39	5,640,455	10.50	5,132,899	1.11	4,614,371 (1778)

Cost estimates for the liquid biopsy test are taken from Morrell *et al* 2025[#], which provides cost estimates for throughputs of 100, 300 and 900 samples per year. Analyses for 40% and 60% availability use the cost for 300 samples per year, and 100% availability uses the value for 900 samples per year. 20% and 80% use the average of 100 and 300, and 300 and 900, respectively.

Costs and outcomes for the Liquid Biopsy columns of each comparison were obtained as a proportional combination of liquid biopsy and pathology costs and outcomes. The comparator for all levels of availability is 100% pathology.

* base case

# Morrell et al. Diagnosing Burkitt Lymphoma in Sub-Saharan Africa by Sequencing of Circulating Tumor DNA: A Comparative Microcosting Study. Value in Health Regional Issues 2025; 48:101113. https://doi.org/10.1016/j.vhri.2025.101113.

ADDITIONAL FILE 7:

Table 3 Scenario analysis: varying duration of risk of relapse

**Years of disease risk**	**Liquid biopsy**		**Pathology**		**Incremental cost (TZS)**	**DALYs averted**	**ICER TZS ($)**
	Total cost (TZS)	Total DALYs	Total cost (TZS)	Total DALYs			
2	10,773,354	9.39	5,640,455	10.50	5,132,899	1.11	4,614,371 (1,778)
3	10,781,446	10.26	5,649,249	11.45	5,132,196	1.19	4,310,769 (1,661)
4	10,789,159	10.87	5,657,439	12.10	5,131,720	1.23	4,172,948 (1,608)
5	10,796,547	11.32	5,665,121	12.57	5,131,425	1.25	4,108,084 (1,583)

Risk of death from BL was extended to 3, 4 and 5 years by adjusting the transition probabilities to reflect predictions of the disease model rather than population age-related mortality. Patients who died were assumed to be in a terminal state in the model cycle in which they die, consistent with the base case, with a terminal disease weight contributing to the Years Lived with Disease (YLD) component of the DALY. These patients also incurred disease-related end-of-life costs during that cycle, again consistent with the base case.

An additional analysis also considered second-line treatment for these late relapsing patients. Treatment costs at relapse during the first years would be captured in our base case data, but later relapses were beyond the study follow-up. Clinical opinion is that it is unlikely that relapsing patients would normally receive second-line treatment in this setting; however, we ran an exploratory analysis where we estimated that 30% of the late-relapsing patients would receive 4 cycles of second-line treatment. Including this cost further reduced the ICER, as the costs increased more in the Pathology arm than in the liquid biopsy arm, so reducing the incremental cost.

ADDITIONAL FILE 7:

Table 4 Further scenario analyses

Scenario	Intervention		Control		Incremental costs (TZS)	DALYs averted	ICER (TZS)	ICER ($)
	Costs (TZS)	DALYs	Costs (TZS)	DALYs				
Base case	10,773,354	9.39	5,640,455	10.50	5,132,899	1.11	4,614,371	1,778
No FN penalty (path)	10,789,564	9.31	5,727,141	10.09	5,062,423	0.78	6,493,108	2,502
Tanzania only	10,105,083	9.17	5,030,020	10.81	5,075,063	1.64	3,096,899	1,193
1-week model cycle	10,757,538	9.44	5,621,771	10.57	5,135,767	1.21	4,578,367	1764

No FN penalty (path): scenario relaxes the assumption that all patients who had a false negative diagnosis for BL by pathology, have the outcomes and costs for advanced stage patients.

Tanzania-only: model parameters from AI-REAL use survival and cost data from only Tanzanian patients (n=46), excluding Ugandan patients.

1 week model cycle: implements a one-week model cycle for the first 2 years, with a revised Weibull model in weeks. Treatment costs are incurred in the first cycle, follow-up costs at the end of each quarter, and end-of-life cost in the cycle in which the patient died. End-of-life disease weights are applied for 3 months as in the base case. After 2 years the model reverts to the base case 3-month cycle.
